# The Challenges of Machine Learning and Their Economic Implications

**DOI:** 10.3390/e23030275

**Published:** 2021-02-25

**Authors:** Pol Borrellas, Irene Unceta

**Affiliations:** Department of Operations, Innovation and Data Sciences at ESADE, Universitat Ramon Llull, ESADE, 08022 Barcelona, Spain; pol.borrellas@esade.edu

**Keywords:** machine learning, AI regulation, algorithmic accountability, welfare economics

## Abstract

The deployment of machine learning models is expected to bring several benefits. Nevertheless, as a result of the complexity of the ecosystem in which models are generally trained and deployed, this technology also raises concerns regarding its (1) interpretability, (2) fairness, (3) safety, and (4) privacy. These issues can have substantial economic implications because they may hinder the development and mass adoption of machine learning. In light of this, the purpose of this paper was to determine, from a positive economics point of view, whether the free use of machine learning models maximizes aggregate social welfare or, alternatively, regulations are required. In cases in which restrictions should be enacted, policies are proposed. The adaptation of current tort and anti-discrimination laws is found to guarantee an optimal level of interpretability and fairness. Additionally, existing market solutions appear to incentivize machine learning operators to equip models with a degree of security and privacy that maximizes aggregate social welfare. These findings are expected to be valuable to inform the design of efficient public policies.

## 1. Introduction

The increasing use of machine learning models [1] is expected to bring several benefits [2,3,4], including improvements in the efficiency of processes, enhancements in the experience of users of products and services, and the development of potentially more impartial decision making systems. A proof of the value added by this technology is the number of fields in which it is already being applied, including healthcare [5], law enforcement [6], financial services [7], employment [8], cybersecurity [9], and autonomous vehicles [10].

Nevertheless, the environment in which machine learning models are trained and deployed is usually complex and introduces several constraints on both the form and the format of the resulting systems [11,12,13]. As a consequence, the increasing use of machine learning has raised concerns regarding, among others, its interpretability [14], fairness [15], safety [16], and privacy [17,18,19]. Even though the technical sources and solutions to these challenges are being intensely studied [20,21,22,23,24,25,26,27,28,29], their economic implications have not been sufficiently examined. This may constitute a problem because these issues can become a barrier to the development and mass adoption of machine learning and, hence, limit the value that this technology can bring to society. Thus, there is a need to fill the gap between data science and economics. Indeed, the unforeseen economic implications of the four issues mentioned above can hinder the maximization of aggregate social welfare that machine learning has the potential to bring. In the context of this paper, we define social welfare as the total utility obtained from a given socioeconomic arrangement or, in other words, as the difference between the benefits and the costs accrued by all the economic agents.

This paper addresses machine learning from an economic perspective and provides new insights into its related concepts. Given the increasing impact of automatic decision making systems on our everyday lives, this work aimed to understand the potential harms derived from the use of this technology and to propose tools to optimally prevent them. In particular, this work focused on determining whether the free use of machine learning maximizes aggregate social welfare or, alternatively, regulations are required. In the cases in which restrictions should be enacted, policies are proposed. For this purpose, literature about both the challenges faced by machine learning and the principles of economic theory were leveraged. The topic is addressed as an economic efficiency problem, i.e., from a positive economics perspective: we focused on “what is” rather than “what ought to be.” Nonetheless, the examined concerns inevitably led to ethical issues that compel normative debates. As Coase and Knight emphasized: “problems of welfare economics must ultimately dissolve into a study of aesthetics and morals” [30]. These additional elements are reserved for future studies.

The main contribution of this work is two-fold. First, it provides policymakers with the state of the art of the challenges faced by machine learning. Second it provides public policy proposals aimed at maximizing the welfare that this technology has the potential to generate. This is expected to serve as the cornerstone for the ongoing debate about algorithmic accountability and machine learning regulation.

This document is organized as follows. Section 2 provides an overview of machine learning, outlines its different methods and algorithms, and discusses use cases. Section 3 provides the context, explains the economic incentives encouraging the use of machine learning and introduces its related concerns. Section 4, Section 5, Section 6 and Section 7 separately discuss four different challenges affecting machine learning. For each one, the sources, effects, and available technical solutions are analyzed. In addition, their economic implications are examined, and public policies are proposed when required. Finally, Section 8 concludes with a summary of our main findings and an outline of future research.

## 2. Machine Learning Overview

Machine learning is a subset of artificial intelligence (AI) focused on the development of mathematical functions, referred to as algorithms, that are able to identify complex patterns in data. Machine learning has the ability to autonomously perform tasks that, otherwise, would be executed by humans or would not be performed at all due to their intricacy.

The most distinctive attribute of machine learning is its ability to learn without being explicitly programmed [31]. Learning in this context is not achieved by manually writing decision rules but by providing algorithms with examples from which patterns are uncovered. Assuming that the chosen examples are representative enough of the studied phenomenon, these patterns are expected to generalize to the rest of the population.

In the machine learning terminology, a model refers to an algorithm that has already been trained or, in other words, that has already been fit to the chosen data. Machine learning models can be classified into three categories on the basis of the method used to perform this fit and, consequently, of the task considered: (1) supervised learning, (2) unsupervised learning, and (3) reinforcement learning.

In supervised learning jobs, the goal is to identify the relationship between a set of input features and a target variable. For this purpose, the model is given access to both the inputs and the outputs through a set of labelled examples. In the cases in which the outputs correspond to categorical data, the resulting models are known as classifiers (e.g., image recognition tasks). Conversely, models that learn to predict numerical outputs are referred to as regressors (e.g., real estate prices prediction). Popular supervised learning algorithms are support vector machines (SVMs), linear and logistic regressions, decision trees, and their improved versions, such as XGBoost. It has to be highlighted that supervised learning is currently the most prevalent type of machine learning. It is used to solve a wide variety of tasks, from skin-cancer classification [6] to the early identification of high value clients in commercial banking [7]. This is why most of the applications and challenges examined in this paper refer to this approach.

In unsupervised learning tasks, data do not include a target variable. Instead, the goal is to identify structures in data to segment them into differentiated groups by analyzing the relationships between the different data points. Some of the jobs that this type of machine learning can accomplish are (1) the clustering of populations, (2) anomaly detections, and (3) association rule mining [32]. For this, algorithms such as k-means and hierarchical clustering are commonly used.

Reinforcement learning widely differs from the other two approaches. Instead of learning from a dataset, reinforcement learning algorithms aim at maximizing the reward defined by an objective function using a trial-and-error approach. Reinforcement learning algorithms receive a task and a set of rules and iteratively try different actions to maximize the obtained reward. These actions are improved on the basis of the feedback received from the objective function. Reinforcement learning could be applied to efficiently manage computer clusters [33], traffic lights [34], robotics [35], and chemistry [36].

Many of the use cases of machine learning outlined above have been possible thanks to the development of artificial neural networks (ANNs), a family of algorithms that resemble the functioning of the brain neurons. These algorithms have been shown to significantly outperform traditional ones in a wide variety of tasks, such as speech recognition and computer vision [37].

Neural networks with complex architectures constitute a machine learning subfield known as deep learning, which could be considered as the state of the art in machine learning algorithmic development. Given that these highly sophisticated algorithms are able to successfully solve the most complicated tasks, they are being increasingly used. This is likely to also be fueled by the aim of companies deploying machine learning to maximize predictive accuracy to address their business needs.

The need to align with these business objectives is just one of the numerous factors that constrain the commercial deployment of machine learning. When used in any company or public institution, machine learning models are constrained by the data they are trained with, as well as by the governance model that controls these data, the different stakeholders that interact with them throughout their lifespan, the software licenses, the regulatory framework, the need to preserve industrial secrecy, and, ultimately, the technological infrastructure that serves the models into production [13]. These factors collectively form the ecosystem of a model and can be understood as constraints that limit the form and the format of the deployed solutions. Often, these limitations result in the use of increasingly complex models.

Unfortunately, the inertia to use more and more complex algorithms comes at a cost. Though these algorithms perform much better than the simpler ones in many tasks, they are also more difficult to interpret [38], which makes it challenging to (1) verify and validate them, (2) provide explanations about their outcomes, and (3) make sure that their behavior is fair. Plus, they are not free from other issues affecting machine learning, such as those related to safety [16,39] and privacy [40].

## 3. The Economic Incentives and Challenges of Machine Learning

Economic agents, especially companies and public institutions, are increasingly employing machine learning solutions in a wide variety of decision-making tasks [2,3,6,9,41,42]. There are a set of economic rationales that fuel this wide implementation of machine learning-powered decision-making tools. Independently of the reasons behind this proliferation of automated systems, the use of this technology has the potential to increase social welfare in many different ways. However, its implementation usually carries side effects. In what follows, we provide an overview of the reasons behind the mass adoption of machine learning by both companies and public administrations, and we introduce the four main challenges that this technology faces today.

The economic incentives that promote the use of machine learning-based tools are manifold and generally vary for public and private organizations.

For companies, the main economic logic is likely to be the aim to gain a competitive edge to maximize profits. Firms are employing machine learning to (1) improve the efficiency of processes, as in the cases of data center and inventory management [2,43] and health treatments optimization [44]; (2) enhance the user experience, as in the cases of granting a safer access to personal devices thanks to facial recognition [45], leveraging targeted ads [46], or providing customized search engine results [47]; (3) reduce the asymmetry of information between parties and palliate the resulting adverse-selection problem, as in the cases of hiring [48] and credit granting [49]; and (4) develop innovative products and services, as in the case of models enhancing speech to the hearing-impaired [50].

If these and other machine learning applications, encouraged by the incentive of companies to maximize profits are commercially successful, they increase social welfare in two ways. First, because their users receive more value in the form of more convenient and personalized products and services. Second, because the derived gains in productivity are expected to eventually lead to lower prices, which increases the purchasing power of customers.

In contrast, for public institutions, the main drivers are expected to be (1) the optimization in the consumption of resources, as in the cases of models aiding police departments to monitor and prevent crime [51] and those detecting tax fraud [52], and (2) the development of more impartial (and fairer) and decision-making processes, as in the case of models used to inform judicial decisions, such as bail, parole, and sentencing [5]. These applications are expected to improve social welfare by facilitating a more efficient use of public resources and a reduction in crime, fraud, and unfair judicial decisions, among other benefits.

Nevertheless, while there are many benefits that come along with the use of machine learning, there are also many potential drawbacks related to this technology. Not in vain, there is a growing trend in the data science field claiming that improving the predictive power of machine learning models ought not to be the sole purpose of the organizations developing and deploying the technology [53,54,55]. In particular, it is argued that the companies and public institutions leveraging machine learning should be held accountable for the behavior and consequences of their models. In other words, that there should be algorithmic accountability. This means that data scientists should not only focus on maximizing the generalization ability of machine learning models but also on making sure that (1) the relevant stakeholders can receive understandable explanations, that they are (2) fair and (3) safe, and that they (4) preserve the privacy of the data used. In brief, four challenges of machine learning are underlined: its (1) interpretability, (2) fairness, (3) safety, and (4) privacy.

In light of this, policymakers are expected to face increasing pressure to limit the use of machine learning to address these issues. Nevertheless, before deciding to regulate the technology and designing laws for this purpose, it is essential to consider the economic implications of these challenges. In this way, efficient public policies can be promulgated.

The following sections separately examine these four concerns affecting machine learning. In all cases, we introduce the challenges and their consequences, and we discuss available solutions under different assumptions. The goal of this examination was to determine whether the use of the technology by organizations in a laissez-faire scenario is expected to be efficient or whether regulations are required to maximize aggregate social welfare.

## 4. Interpretability

A relevant problem suffered by machine learning models is the difficulty to understand how they make decisions. This is the result of their inherent complexity: machine learning models are able to find sophisticated data patterns that even expert statisticians cannot understand. This means that interpreting the outputs of these models is not always an easy task.

In an effort to overcome this issue, a research subfield known as explainable artificial intelligence (XAI) that concentrates on the development of techniques to make machine learning models interpretable has thrived in the past few years [14,56]. In this context, a machine learning model is deemed interpretable as long as the relevant stakeholders can obtain and understand the reasons behind its decisions [57], i.e., if they receive proper explanations, as defined by [58].

If stakeholders do not receive proper explanations, it is difficult to precisely verify and validate the functioning of models [59]. Verifiability entails rejecting the hypothesis that a model is basing its decisions on data artifacts and controlling that it can reasonably cope with unexpected outliers. Validation involves testing that the model is able to generalize sufficiently well. This implies that the lack of interpretability of most machine learning models represents both a critical hurdle for algorithmic accountability and a barrier to some of their applications.

### Discussion and Proposed Solutions

A lack of interpretability can generate a lack of trust on the outcomes of models. This is particularly worrying in safety-critical domains [60,61] that call for trust on the part of both the expert users of models, such as physicians, and engineers, and the individuals affected by their decisions, such as patients and the inhabitants of a building. A wrong decision or a good decision for the incorrect reasons can endanger human beings and generate an economic loss. This, in turn, could affect the demand: if sufficient trust is not provided, customers may opt not to use these models. In general, this attitude of the demand side of the market incentivizes organizations to optimally fund research to develop interpretation techniques with the aim of identifying and removing biases and generating trust [62]. Consequently, the incentives of all the involved stakeholders are expected to be aligned and lead to the maximization of aggregate social welfare.

On top of this, a lack of interpretability also deprives the affected parties of the reasons behind a potentially sensitive decision. From an ethical point of view, it could be perceived as immoral to let non-interpretable machine learning models make decisions that affect people without informing them about the reasons behind the choice or the ways to contest it and obtain a different outcome in the future. From this normative perspective arises the so-called right to explanation. The recognition of this right would compel public institutions and private companies to justify the decisions generated by any machine learning model and ensure their fairness and legality [63]. Nevertheless, the promulgation of such a right would also entail that some applications for which sufficiently good explanations can only be provided by using simpler models with a much lower predictive power would be dismissed. Additionally, given that generating explanations has a cost, all the use cases in which the marginal cost of producing an explanation is larger than its marginal benefit would, presumably, also be disregarded. Moreover, it could slow down innovation in the field, given that some of the current and future commercial applications would be unfeasible or costlier and take a longer time to market, which would likely deter funding.

Thus, the effect on aggregate social welfare of promulgating such a right would be negative unless the cost that users suffer by not obtaining explanations is larger than the benefits of using the models. This hypothesis, however, seems unreasonable given that, if individuals valued the receipt of explanations that much, and assuming that they make bounded rational choices that optimize their utility given the alternatives available, none of the applications employed by non-monopolistic companies would be commercially successful. Again, the demand-side market power seems to ensure the optimal level of interpretability and the maximization of aggregate social welfare.

Still, in the case of machine learning models employed by companies with an abnormal market power, customers may lose their demand-side market power, which can lead to a suboptimal welfare equilibrium because firms do not have incentives to adapt their offerings to the preferences of clients. Similarly, public institutions, which have the power to force citizens to use a model, may provide an insufficient level of explanations if a minority of voters without the sufficient bargaining power value them so much so as to compensate the social cost that enforcing a right to explanation would entail. In these specific cases, enforcing a right to explanation could optimize the aggregate social welfare. Even so, it must be noted that estimating such utility functions seems unfeasible in most cases.

Finally, a lack of interpretability may also impede properly determining whether a machine learning model was the cause of any harm. This damage could be the result of faulty or biased machine learning models. In these situations, common legal norms, such as tort law and anti-discrimination laws, usually establish that the harmed party has to be compensated by the offender when a causal link can be reasonably proven, which constitutes a fault-based liability policy. However, proving such a causal link may be difficult and costly when non-interpretable machine learning models are involved.

To properly analyze this issue, it is useful to classify the damages that machine learning models can produce according to their nature. Two categories are proposed: (1) discrimination-related harms, which are those arising from the unfair treatment of certain population groups, and (2) non-discrimination-related harms, which are the result of fair but faulty machine learning models. Given the significance and complexity of discrimination-related damages, this analysis is separately performed in Section 5.

Non-discrimination-related harms refer to the damages caused by machine learning models that are deemed fair but that are faulty in any of the following two ways: (1) the model suffers a non-expected behavior while deployed in the wild, or (2) the model makes a mistake which is expectable given the performance metrics obtained during the training phase. Legally, these cases can be treated in very different ways. Fault-based liability is the most common one, but a strict liability policy or a reversal of the burden of proof (in which the parties in charge of monitoring the functioning of the models and benefitting from their use are required to prove the lack of causality), could also be applied.

If the fault-based liability regime is applied, the burden of proving causation or fault relies on the plaintiff side. In the cases in which the origin of the harm is not clear (e.g., an autonomous car is involved in a crash with other vehicles involved), the complexity to prove causation due to (1) the lack of the interpretability of machine learning models, (2) their possible continuous retraining, and (3) their interaction with different pieces of hardware and software would significantly reduce the chances of success of any claim for compensation [64].

This would decrease the amount of claims and incentivize the generation of a suboptimal amount of damage to users and third parties. In the case of the users of the product, this inefficient allocation of harm would discourage consumers from embracing this technology. This reaction from the demand side of the market incentivizes the companies leveraging machine learning models in safety-critical domains to provide a sufficient level of interpretability to validate and verify their behavior and improve their safety.

In the case of harm borne by third parties, who may not have the ability to put pressure on the organizations, the burden of proof can be reversed so that developers, distributors, and operators are the parties required to prove that their product did not cause the harm.

In a different manner, in the cases in which the main origin of the harm is clear (e.g., an autonomous car crashes on its own), it is easy for victims to prove causation, thus incentivizing manufacturers to develop interpretable models to validate and verify their behavior and improve their safety.

Nevertheless, it could be complex, if it is possible at all, to determine who is the real originator of the damage even when the general cause is clear. For instance, it might be difficult to identify which specific component, potentially manufactured by a company different from the seller of the final good, caused the accident. This could occur because the device causing the harm can contain many tightly interdependent technologies from different producers and with different operators. In this scenario, the common solution is that all parties are jointly and severally liable, which is not desirable for the tortfeasors who did not cause any harm [65]. This complicates an efficient allocation of costs between the different involved manufacturers. Nonetheless, these parties are expected to efficiently allocate costs in the case that the final product causes harm ex-ante, during the contractual negotiations, based on the expected performance of the different components, including machine learning models. For this reason, the developers of machine learning models are also incentivized to provide an optimal level of interpretability in this scenario.

Alternatively, a general strict liability policy (which is only commonly used in some specific situations) could be applied. In this case, proving causation would not be required. Instead, victims would just need to prove that the risk triggering strict liability took place [50]. This risk could be as straightforward as the machine learning model being involved in the incident. This policy would guarantee the right to an effective access to justice, given that the chances of not receiving compensation when the case is lawful would be minimal. Moreover, the potentially high transaction costs associated with proving causation would be removed [66]. At the same time, though, this situation would require the parties involved in the development and deployment of the machine learning models to pay a suboptimal amount in compensation because they would be liable even when they did not cause the harm. This would likely deter the advancement of the technology and cause an increase in prices to compensate for the additional risk and costs, which would slow down the adoption of machine learning-powered solutions. Consequently, this liability regime should be applied only in very specific cases, such as those in which the harm generated by the model can be significant and impossible to compensate [67].

Similarly, another policy could be reversing the burden of proving causality or fault in all cases so that developers, distributors, and operators (the parties in charge of monitoring the functioning of models and benefiting from their use) are in charge of demonstrating that the damage was not caused by a malfunction in their product or service. The benefit of this liability policy is that these parties are likely to be the cheapest cost avoiders to prove causality because they have direct access and knowledge over the technology. In addition, under this criterion, they would have incentives to design more transparent and interpretable machine learning systems to be able to prove the no-causation. Plus, victims would be discharged from proving causality, something at which they would likely fail or have to incur unduly costs otherwise [64]. Still, in this scenario, the organizations involved in the development and deployment of the machine learning model may have to bear an inefficient amount of compensation if they are unable to prove the lack of causality, though to a lower degree than under a strict liability policy. They would also have to incur additional costs to make their models more interpretable than optimal to be able to avoid the additional compensation costs arising from the lower level of care expected by users, who would be incentivized to take less care than in the case of fault-based liability.

In conclusion, the most efficient liability regime, the one maximizing aggregate social welfare, is expected to be a regular fault-based liability with some exceptions in which the burden of proof is reversed and others in which strict liability is applied (see Appendix A). The reversal of the burden of proof policy is expected to increase efficiency in those cases in which the victims would have likely failed or would have had to incur unduly costs to proof causation because of the substantial asymmetry of information between the parties [67]. Similarly, the strict liability regime is expected to improve efficiency in those cases in which machine learning-powered products are operated in non-private environments and can cause significant and impossible-to-compensate harm even when the agent acts with due care [67].

Lastly, it is clear that the development of better and easier-to-use interpretation techniques is expected to increase the aggregate social welfare by (1) “unlocking” the machine learning applications that require reliable explanations, (2) attracting users who would otherwise be reluctant to use the technology, and (3) reducing the transaction costs and inefficient allocation of loss in the case of incidents in which models are involved.

## 5. Fairness

Machine learning models fall under the category of actuarial decision-making methods. In contrast to the clinical method, which is wholly based on the human processing of information (which is affected by cognitive distortions [68], attitudes, and beliefs that can lead to discrimination [69]), the actuarial approach is set up on empirically established relations coded into mathematical formulas that produce automatically decisions. Research over the years has shown that the actuarial approach systematically leads to more accurate decisions [70,71,72], it has a limited human intervention, and it is a formal process that can ensure the non-employment of protected features. Based on this, machine learning-powered decisions could be perceived as the ideal way to eradicate unfair decisions produced by the flawed subjective judgement of humans. Nevertheless, machine learning is not free from reproducing this same behavior and can even generate a false sense of objectivity by making predictions based on data. Indeed, if the performance of machine learning models is not evaluated in terms of fairness, even well-intentioned applications might give rise to objectionable results [15].

This paper considers a machine learning model to be fair if its most relevant error rate, such as accuracy, recall, or precision, does not systematically differ between the involved population groups, especially between the protected and unprotected ones. The reason for not being able to optimize all the types of error rates is that it is mathematically impossible that different groups have more than one equal metric if the prevalence of the event is different between them [73]. A required but not sufficient condition to comply with this criterion is that the decisions of the model solely depend on relevant features to the outcome of interest, i.e., that the differential traits among population groups are not considered as long as they are not relevant for the task at hand. Any machine learning model not meeting this definition is deemed biased.

Machine learning models can suffer biases that can result in unfair outcomes if (1) the data on which they are trained are biased or (2) the models themselves are poorly trained.

The data on which models are trained are likely to be biased because (1) human intervention affected their representativeness, (2) current data patterns may have changed or are socially undesirable, or (3) minority groups do not have enough representation. The training dataset is frequently a collection of past events that had a degree of human intervention in the selection of the sample and/or the labelling of the dataset. For instance, a credit scoring model that determines who is eligible for a mortgage is usually trained with historical data that are sampled and labelled by the individuals who were in charge of determining who was granted a secured loan. These people based their decisions on a subjectively-judged set of variables, which affects both the composition of the sample and its labelling. Given that individuals suffer cognitive distortions and have beliefs that affect their actions, these past examples may reflect historical prejudices and cultural stereotypes over certain population groups and demographic inequalities that can lead to biased models that work as reinforcement loops of the status quo [15].

Additionally, because historical data are, by definition, collected in the past, they are prone to become useless in the present if there are changes in the underlying data patterns. In other words, variables that were good predictors of an outcome in the past may lose their predictive power over time. This phenomenon, known as concept drift [74], can affect both a model’s performance and fairness. Following the previous example, since the financial institution collected data from a specific past time window that had its own structural idiosyncrasies, the representativeness of the sample will be damaged if the macroeconomic outlook evolves over time and develops previously unseen characteristics. Moreover, if the prevalence of the event being evaluated among the different population groups changes after the data collection, the performance and fairness of the model will be negatively affected too. For instance, if in the past, only certain groups with differentiated profiles had access to financial services or if there was a self-selection of candidates, the machine learning models fed with these data are likely to face problems to accurately classify new customer profiles. The same happens if the current prevalence is the result of widespread biases and is socially undesirable, such as the underrepresentation of women in Science, Technology, Engineering, and Math (STEM) jobs [75]. This historical difference can hinder their access to these types of jobs if a biased machine learning model is employed to select applicants [41]. This source of bias is known as stereotype mirroring.

In the last place, training datasets may also count with an insufficient amount of data points representing minority groups. This is known as sample size disparity and can result in higher error rates for these groups if they are significantly different from the majority group for the predictive task at hand [15]. This happens because the performance of models for each group depends on the amount of data used to train them. It has to be highlighted that, in this case, the reason for the underrepresentation may not be related to societal biases but to the historical or natural lower prevalence of some population groups that constitute minorities, such as children. Thus, if such a problem is not taken into account when deploying a model, minority groups can suffer systematically lower rates of accuracy in decision-making, which can be deemed unfair [76].

Second, even in the case in which the data are representative, a poorly trained model can produce biased outcomes due to (1) technical limitations and (2) the use of correlations to build data patterns.

State-of-the-art algorithms can find it difficult to detect and characterize certain features that are naturally present in the real world. For instance, face recognition models appear to systematically perform worse at identifying female and dark skin faces [77]. This could partially be the result of the historical optimization of image technology to capture light-skins [78]. Therefore, the use of unbiased data to train a machine learning model does not imply fairness because error rates can widely differ between population groups anyway.

Another problem is that, at the moment, machine learning identifies patterns are based on correlations, not causal relations. Consequently, poorly trained models are likely to capture both signal and noise in the form of confounding variables. This implies that they may also rely on protected features, such as ethnicity and gender, even when they are not explicitly included. This can happen because these variables are likely to be redundantly encoded in any sufficiently rich set of features [76]. For instance, zip code, income, and ethnicity are known to be highly correlated in some countries such as the US [15]. This can result in erroneous decisions that hinder the access to certain resources to the affected parties even when models are well-intentioned and built with apparently unbiased training datasets.

Thus, the lack of fairness that some machine learning models show can be considered a socio-technical problem, given that it can arise from both human-generated discrimination and technological limitations. The harmful effects of biased machine learning models can be grouped into two types: (1) allocative harm and (2) representative harm [79].

Allocative harm refers to machine learning models that withhold resources or opportunities from certain groups of people on the basis of non-relevant features, e.g., a model that systematically rejects granting a mortgage, loan, or insurance to women or non-Caucasian people for this reason. This kind of harm has immediate effects; it is easy to quantify and has a transactional nature [79]. These characteristics can make its effects very salient, especially when it results in disparate impact (unintended disproportionate adverse impact on a particular group) in critical contexts, such as pretrial detentions [80] and employment [41].

In contrast, representative harm refers to machine learning models that reinforce the subordination of some groups based on ethnicity, social class, or gender. Illustrative examples of this kind of harm are the Google Photos image classifier tagging dark-skinned people as gorillas [81] or the propensity or Google’s search engine to display criminal-background-check ads when Afro-American-associated names are queried [82]. While the former example constitutes a pure representative harm because it denigrates people, the latter is both representative and allocative because such occurrence is hypothesized to negatively affect employers’ judgement of candidates with such names in addition to reinforcing ethnic stereotypes. It has to be highlighted that this type of bias has longer-term effects than the allocative one because it has the potential to affect people’s attitudes and beliefs and because its effects are more difficult to identify and quantify [79].

Solving these problems from a technical side is challenging due to (1) the poor interpretability of algorithms and (2) the complexity to remove bias from training datasets. Taking this into account, all the current research directed at improving the interpretation and understanding of the decision-making logic of models [14,24,56] is expected to contribute to solving the fairness issue by allowing for its easier detection.

### Discussion and Proposed Solutions

As explained, even well-intentioned machine learning models can result in unfair outcomes. The immediate questions are then whether the companies and institutions employing this technology have the incentives to develop and employ the fairest possible models and, if so, whether these models are fairer than the best alternatives available. It is also essential to infer whether this “algorithmic fairness equilibrium” maximizes social welfare.

To achieve this purpose, it is required to analyze (1) the current incentives of the organizations, (2) the fairness and social welfare that they are likely to induce, and (3) whether the resulting machine learning models are the best available alternatives. Given their different nature and incentives, companies and public institutions should be separately examined.

Companies are incentivized to only develop and deploy those machine learning models whose return on investment is positive. This, in turn, is going to depend on (1) the opinion of its final users, customers, and parties affected by the model; (2) their consequent behavior; and (3) the reaction from competitors and the public opinion, which may have the ability to harm the firm’s brand image and reputation. Thus, companies are expected to only deploy models which sufficiently satisfy these stakeholders in terms of value added and algorithmic fairness, even when the development of fair models comes at the cost of higher time to market. Given that firms strive to optimize resources, this demand-side market solution can be assumed to maximize social welfare in the absence of negative externalities. Still, the maximization of social welfare does not imply that the machine learning models are deemed fair because (1) minorities may not have enough market power to affect companies’ decisions and (2) models may be mirroring current stereotypes or inequalities with insufficient social rejection.

In the first case, the solution is the use of anti-discrimination laws. Anti-discrimination laws, which are present in most of legislative bodies, forbid the discrimination of citizens on the basis of a set of protected characteristics, such as ethnicity and gender. These rules apply to human-based decisions and machine learning-based ones. This way, any person who feels discriminated by any machine learning model is able to legally prosecute the company that is deploying the suspicious decision engine. If the model is proved to be biased on the basis of the most appropriate definition of fairness according to the judge, the firm faces a penalty.

This way, the rights of minorities are protected, and companies, anticipating this situation, are incentivized to take algorithmic fairness into account from the beginning of the development of the model. Nevertheless, this solution is expected to be problematic nowadays because of (1) the lack of interpretability that some models may present, which makes their assessment complex; (2) the possible lack of understanding of the technology by non-expert juries; and (3) the need to protect trade secrets.

In the cases where models mirror present stereotypes, companies may choose to tweak algorithms to remedy the situation and benefit from an improvement of their brand image and reputation. For instance, Google published a list of principles followed when developing and deploying artificial intelligence technologies, which includes avoiding the creation or reinforcement of unfair biases [83]. If it is not the case, the change in paradigm may be forced by the direct intervention of the representative institutions of the state or by the indirect effect of private institutions pushing for a societal change, which may affect consumers’ attitudes and beliefs and put more pressure on companies.

In sum, private firms are expected to develop machine learning models that (1) maximize social welfare (in the absence of negative externalities) and (2) try to be the fairest possible in use cases in which the different stakeholders exert enough pressure.

Unfortunately, algorithmic fairness is found to be achieved at the cost of predictive accuracy in at least some use cases [84,85]. This complication, known as the accuracy–fairness trade-off, has two crucial implications: (1) companies may have incentives other than the cost to avoid tackling the algorithmic fairness issue, particularly in those use cases with limited public scrutiny, and (2) efforts to improve the fairness of models are likely to imply a reduction in the economic value they generate.

This makes the efficient functioning of the anti-discrimination law procedures more critical, as too slow or costly mechanisms can deter discriminated individuals from pursuing a legal claim. Furthermore, it raises another key concern: modifying algorithms to comply with a specific definition of fairness is likely to lead to a smaller increase in social welfare. In other words, the accuracy–fairness trade-off can also be considered a social welfare–fairness trade-off. At this point, the discussion is purely normative. Thus, the different stakeholders should decide whether it is reasonable and ethical to systematically penalize some population groups to favor the aggregate social welfare or, in contrast, fairness should prevail despite leading to a worse equilibrium. Since machine learning applications are highly diverse, including in their social implications, the best solution may be to separately perform a cost–benefit analysis of each case and let judges decide the optimal equilibrium in the relevant applications. There is not a one-size-fits-all solution.

In the case of democratic public institutions, their ability to leverage machine learning is limited by the body of legislation promulgated by the parties conforming a majority in the parliament, whose main incentives are expected to be the retention and, possibly, expansion of their power. For this, they depend on the public opinion, which is critical to obtain sufficient votes to achieve their objectives. In this situation, the opposing parties can be considered their competitors. Thus, these organizations can be expected to deploy machine learning models that do not lead to a loss in popular support, which would jeopardize their position of power. In other words, the majority of voters are the ones influencing the deployment of models by democratic public institutions. For instance, a government is not expected to deploy a mass surveillance system powered by machine learning, which could not only contribute to safety but also to political prosecution and a loss of privacy if the majority of voters are not predicted to support the idea.

Taking this into account, and assuming the absence of corruption, democratic public institutions are expected to use models that, at least, contribute to an increase in the social welfare of a fraction of the population. Nevertheless, this does not imply that the aggregate social welfare is maximized because, in contrast to the case of companies, the effects on citizens of these machine learning models are likely to be unavoidable. Following the previous example, if a limited majority of voters were in favor of deploying a system of mass surveillance because they value the increase in safety but the remaining citizens perceive it as a massive loss in privacy, the resulting net effect on aggregate social welfare would be negative.

This illustration also points out that democratic public institutions may not have the incentives to use only unbiased machine learning models since minorities do not have, by definition, the sufficient demographic weight to enforce fairness by themselves. Again, to ensure the deployment of sufficiently fair models, it is required to have anti-discrimination laws. Given the influence that the executive and legislative bodies are likely to have, an independent judicial system and strong constitutional guarantees are key to enforce anti-discrimination mechanisms. Still, this solution may not properly work for the three stated reasons in the case of the models that are deployed by companies: (1) the lack of interpretability that some models present, (2) the possible lack of understanding of the technology by non-expert judges, and (3) the need to protect trade secrets.

The first two points may deter discriminated citizens from pursuing a legal claim because these factors are expected to decrease the probability of successfully proving the unfairness of a model, which diminishes the expected benefit of performing such action. The third point has two important indirect effects, as forcing companies to publicly disclose trade secrets has the potential to (1) damage their incentives to invest in such innovative technologies because any competitive edge would be lost, which reduces technological progress, and (2) undermine the security of models by disseminating sensitive information to third parties about how to game it (see Section 6).

To palliate these problems, two policies are proposed: (1) the publication of industry standards and good practices regarding algorithmic fairness and (2) a modification of anti-discrimination law procedures to accommodate the specificities of machine learning models (see Appendix B).

Lastly, to assess the algorithmic fairness of models, it is essential to consider the best available alternatives, given that the technical limitations to build completely fair models can make this objective unattainable at the moment. In other words, assuming that fairness is a difficult-to-achieve objective, it is reasonable to determine the social desirability of machine learning models on the basis of the performance and the cost of the available alternatives, such as human-based decision making. This exercise, which is expected to be naturally performed by the different stakeholders, from final users to judges, should consider the specificities of each use case.

In conclusion, in a laissez-fair scenario combined with well-functioning anti-discrimination laws, the optimization of the social welfare–fairness trade-off is expected to protect minority groups in relevant cases and to keep evolving as a function of the degree of concern for the algorithmic bias of society, which has the ability to put pressure on the need for fairness. Still, given that the fairness metric to be optimized is a subjective decision, it is likely to differ depending on the stakeholders determining it, as each stakeholder may have diverging objectives. Thus, it is likely that not all stakeholders agree on what constitutes a fair model.

## 6. Safety

Machine learning models have been shown to be susceptible to adversarial input perturbations that can alter both the produced predictions and explanations [16,86]. Adversarial examples are inputs that are specifically perturbed to mislead machine learning models in human-imperceptible ways. For instance, in the context of computer vision, this kind of attack could affect the classification of traffic signs and classify a stop sign as a speed limit one, which could cause accidents. This lack of safety is usually not caused by the presence of negligence during their development. Adversarial attacks can succeed without the attackers needing to have any substantial knowledge about the inner functioning and characteristics of the model as long as they can (1) query it and obtain the output associated to each input [87,88] or (2) modify the composition of the used training dataset [16].

This type of attack can be classified based on the phase in which they affect the functioning of machine learning models [16]. Malicious techniques that mislead models during the testing time are known as evasion attacks. In contrast, the ones that fool them during the training time are known as poisoning attacks.

The specific goal of evasion adversarial attacks is to misclassify specific inputs without corrupting the model’s inner functioning. These attackers may be interested in (1) swapping a class for another specific one or (2) just misclassifying a class, regardless of the wrong attributed label [16].

In a different way, poisoning adversarial attacks affect the behavior of machine learning models in two ways. First, they can aim at compromising the normal functioning of the model and, potentially, cause a denial of service. This depends on whether the aim of the attackers is to cause generic errors or specific misclassifications, as well as on the number of perturbed inputs successfully included in the training dataset [16]. Jagielski et al. [27] demonstrated the large effects that a small share of perturbed data points could produce by performing poisoning attacks over three datasets containing health, financial, and housing data. In a health-related case, the presence of 8% of perturbed inputs is able to change the dosage of a drug by 75% for half of the patients.

Given the broad implications of this problem, a subfield of machine learning focused on developing solutions has appeared. This research field is known as adversarial machine learning. Despite the progress achieved so far [26,89,90], new adversarial attack algorithms are being developed [86,91]. Consequently, the nature of this problem indicates that this threat is going to resemble the “arms race” experienced in software, in which security advances are followed by attack improvements and vice-versa.

### Discussion and Proposed Solutions

This lack of robustness in the outputs of machine learning models makes it risky to use this technology in safety-critical domains, such as autonomous driving [91], because harm can be generated to users and third-parties. Consequently, this problem is likely to delay or even impede the mass-adoption of several machine learning applications that have the potential to generate value for society. In other words, this threat limits the increase in aggregate social welfare that the technology has the potential to generate.

In the current scenario, the restriction in the use of the technology in the domains in which sufficient security is not guaranteed would arise from (1) the rejection to use these machine learning-powered products and services by consumers or voters and (2) the deterrent effect produced by the compensations imposed by tort law if damages are caused [92]. This means that any organization aiming to successfully deploy a model is forced to invest resources to ensure the minimum safety requirements for its commercial success and social acceptance. Thus, market demand and legislation already provide strong incentives to make these machine learning applications sufficiently robust.

Nevertheless, what is the optimal level of risk? A zero-risk scenario looks unfeasible in the near future. Hence, the optimal level of risk will be that in which the marginal cost of increasing the security of models is larger than the benefit it would bring. Consequently, as long as the marginal benefit of developing better reactive and proactive defensive techniques is greater than their marginal cost, research in this field is expected to be financed not only by public means but, especially, by private funds. Companies are the organizations with the strongest incentives to fix the security threats because they are the ones likely to financially benefit the most from it. For instance, no vehicle manufacturer or technology company is expected to systematically launch autonomous cars and machine learning-powered voice assistants, respectively, if they cannot ensure the tacitly required robustness to adversarial attacks.

As a result, there is no need for any policy proposal: the current incentives appear to be appropriate to make machine learning models with the potential to increase the aggregate social welfare optimally safe in the shortest amount of time.

Lastly, it is important to highlight that, to prevent attackers from gaining access to the functioning of machine learning models and to avoid increasing their vulnerability, which would slow down their deployment and adoption, it is essential that trade secrets remain confidential even when models are audited to prove fault or the presence of biases.

## 7. Privacy

Machine learning models are also inherently vulnerable to attacks aimed at capturing the training data of the system without requiring negligent development [40]. The intrinsic functioning of machine learning models may lead to the unintentional leakage of information about their training data through their parameters and outputs [40]. Though they are more vulnerable when their internals are known by adversaries, these attacks have also been shown to be possible in black-box scenarios [18].

In most cases, the attackers’ aim is to illicitly gain access to information about the model and the data used to train it, which is likely to be valuable for mischievous activities, such as frauds or the performance of sophisticated adversarial attacks [16]. Consequently, these privacy-related attacks can enable or facilitate security-related attacks, such as evasion ones, because they allow for the training of the surrogate models employed with the same or similar data to the target one [16].

Privacy-related attacks can be classified according to their motivation and modus operandi. This paper distinguishes three types: (1) model-inversion attacks, (2) membership-inference attacks, and (3) malicious machine-learning-as-a-service attacks.

In a model-inversion attack, the aim of attackers is to uncover the values of sensitive features associated with a specific identity in the training dataset. This could be as simple as revealing the genetic markers of patients [93] or as complex as reconstructing the faces of users of a biometric authentication system [40]. In a different manner, membership-inference attacks aim at determining whether a specific data record was part of the training dataset used to train a model [18]. Lastly, private training data can also be endangered if malicious training algorithms from a machine-learning-as-a-service provider are used. This has been proven to be feasible even when the target model is trained and hosted on trusted cloud platforms that provide isolated environments and ensure that the model does not communicate with the internet except to transmit its outputs, which prevents attackers from directly accessing the training dataset [19].

Several technical solutions exist to prevent these three types of attacks, such as k-anonymity [82,94], differential privacy techniques [28], and the use of synthetic data [95]. Nevertheless, most of them imply the perturbation of the training dataset or the parameters of the models, which eventually damages their performance. As a result, a privacy–accuracy trade-off arises. This phenomenon taxes their use in real applications [17,18,19,40,96].

### Discussion and Proposed Solutions

If the possibility of experiencing privacy-related attacks is negatively perceived by users and consumers, the development and mass-adoption of machine learning is likely to be hindered. First, if a sufficient level of security cannot be provided with the available technical solutions, commercial success is not possible and the benefits provided by models are not exploited. Second, in the cases in which the cost of the measures to increase the safety of models is larger than the additional benefits of these, the model becomes unprofitable.

It is also important to highlight that, if data subjects highly value their privacy, they are expected to be reluctant to cede their data even when the machine learning models that their data enable are beneficial for them. If this was the case, the participation in the generation of training datasets would resemble a public good problem because individuals would be incentivized to free-ride: seek the benefit of using the models without incurring the costs of being in the dataset. This would lead to an under-provision of data and, as a result, to less and worse performing models, which reduces their value added.

Thus, the lack of privacy of machine learning models has the potential to delay and restrict the mass-adoption of valuable machine learning applications, thus reducing the aggregate social welfare gain that the technology has the potential to generate. This is especially expected to happen in domains that require the intensive use of sensitive data, such as financial services, healthcare, and authentication tasks. Notwithstanding, this issue is also relevant in less critical applications, such as social networks and search engines, given that the right to privacy can be perceived as fundamental to ensure human dignity even when non-sensitive data are produced and potentially misused [97].

In this scenario, are the organizations developing and deploying machine learning models incentivized to invest the optimal amount of resources to provide the degree of privacy that maximizes aggregate social welfare? Yes, they are incentivized to do so to avoid (1) not being able to collect and exploit enough data, (2) being rejected by consumers, (3) losing their competitive edge to the parties being able to capture their datasets who could more precisely replicate the attacked models, (4) suffering stronger evasion adversarial attacks [16], and (5) experiencing reputation-related crises arising from these malicious interventions. Additionally, but these organizations could also face penalties imposed by data protection laws, such as European Union’s General Data Protection Regulation (GDPR), in the case of data breaches [98].

Consequently, the companies and public institutions using machine learning are highly incentivized to invest the optimal amount of resources to take the appropriate defensive measures.

These incentives do not apply in the case of malicious machine-learning-as-a-service attacks because they are malevolent on purpose—they are designed to capture the training data of their users. In this case, the main vulnerability is the asymmetry of information between the users and the suppliers of such services. Given that users cannot identify which purveyor is malevolent and which is not, even after using their services, these platforms will be less used the more known this threat is. As a result, at some point, market solutions to the problem are expected to arise. Benevolent machine-learning-as-a-service platforms are expected to signal their quality to users by, for instance, being certified as trustful suppliers by independent third parties. Additionally, the organizations that currently offer these services as part of a wider portfolio, such as Amazon, Google, and Microsoft, are also less likely to perform these practices, as their reputation is at stake. Moreover, if data privacy laws are enacted, malicious companies would also face legal liability, which reduces their incentives to perform these attacks.

In sum, market mechanisms are expected to properly align the incentives of the parties developing and operating machine learning models and machine-learning-as-a-service platforms. In other words, if privacy-related threats are perceived as too severe by users and consumers, machine learning operators are incentivized to invest resources to optimally meet the market demands. These incentives, in the absence of negative externalities, lead to the maximization of aggregate social welfare. Consequently, no additional regulation appears to be necessary. In fact, harsh machine learning privacy standards could even generate inefficiencies and make some applications unprofitable or worthless given that they entail (1) monetary costs that may not be sufficiently valued by consumers and, potentially, (2) a reduction in performance. As a result, the promulgation of laws such as the GDPR may not be desirable from a positive economics point of view because, in its absence, consumers are expected to naturally select the organizations that comply with their privacy requirements.

## 8. Conclusions

This paper has examined four challenges faced by machine learning and their economic implications. Namely, the issues analyzed are the potential lack of (1) interpretability, (2) fairness, (3) safety, and (4) privacy of the technology. The aim was to determine, from a positive economics point of view, whether the free use of machine learning maximizes aggregate social welfare or, alternatively, regulations are required. In the cases in which restrictions should be enacted, policies have been proposed.

It has been found that current tort and anti-discrimination laws should be adapted to the specificities of machine learning to incentivize an optimal level of interpretability and fairness. Regarding tort law, a combination of fault-based and strict liability and the reversal of the burden of proof under some circumstances has been proposed (see Appendix A). In the case of anti-discrimination laws, two policies have been proposed: (1) the publication of industry standards and good practices regarding algorithmic fairness and (2) the modification of the current legal procedures to ensure that juries have the appropriate information and knowledge to examine these kind of cases (see Appendix B).

In contrast, existing market solutions appear to encourage machine learning operators to equip models with a degree of security and privacy that maximizes aggregate social welfare. This happens naturally because machine learning operators are incentivized to invest resources to optimally meet market demands. In other words, if security and privacy-related threats are perceived as too severe for users and consumers to adopt the models, operators are forced to find solutions. Consequently, there is no need for any additional policy; the current incentives appear to be the appropriate to make machine learning models optimally safe and privacy-preserving.

In sum, the recommended public policies, together with the power of demand-side market solutions, are expected to maximize aggregate social welfare without the need to explicitly limit any machine learning application or promulgate a right to explanation and to contest. Tort and anti-discrimination laws and consumers’ and voters’ pressure are expected to sufficiently align the incentives of the organizations developing and operating machine learning models so that both present and future use cases generate more value than costs.

Still, two considerations should be made regarding the optimization of fairness. First, given that the fairness metric to be optimized to deem whether a model fair is a subjective decision, it is likely to differ depending on the stakeholders determining it, as each stakeholder may have diverging objectives. Thus, it is likely that not all stakeholders agree on what constitutes a fair model.

Second, in the case of machine learning models employed by companies with an abnormal market power, customers may lose their demand-side market power, which can lead to a suboptimal welfare equilibrium because firms do not have incentives to adapt their offerings to the preferences of clients. Similarly, public institutions, which have the power to force citizens to use a model, may provide an insufficient level of explanations if a minority of voters without the sufficient bargaining power value them so much so as to compensate for the social cost that enforcing a right to explanation would entail. In these specific cases, enforcing a right to explanation could optimize the aggregate social welfare. Nevertheless, estimating such utility functions seems unfeasible in most cases.

Lastly, it is important to highlight that, in general, the objective of regulations should not be to minimize the risk of damages and attacks or to ensure that all models are interpretable and fair, which entails an opportunity cost, but to guarantee that their aggregate net effect is positive and the largest possible [30]. It is clear, then, that with these measures in place, some of the deployed models will remain poorly interpretable, biased, and not robustly safe and privacy-preserving. Nevertheless, these cases are only going to be those that the affected parties do not consider important enough to trigger legal claims or boycotts.

A key lesson from this study is that there are no easy solutions to the challenges that machine learning faces. The reasons for this are that these issues are inherent to the technology itself, and that the technical solutions publicly available at the moment are not able to completely resolve them. Additionally, the different trade-offs that exist among the performance, interpretability, fairness, security, and privacy of models make solving these challenges even harder. For instance, the need to protect models from evasion adversarial attacks and to keep training data safe may prevent operators from providing comprehensive explanations about their functioning and outputs. These facts, together with the wide variety of use cases in which machine learning is applied, imply that there is not a one-size-fits-all solution. Consequently, any new public policy should be flexible enough to simultaneously avoid over-harming the competitiveness of the technology and to allow for efficient judicial decisions in case of litigation. It is also important to bear in mind that, given that the use cases enabled by the technology are expected to keep evolving, regulations are likely to require further upgrades in the future.

We expect these findings to be valuable to inform the design of efficient public policies. This is particularly relevant given that public institutions around the world are starting to study the ways to regulate machine learning [99,100]. These laws, if poorly designed, could hinder the increase in social welfare that this technology has the potential to generate. Of course, it is reasonable to expect that these legislative debates include other social concerns related to the mass adoption of machine learning, such as ethics and inequality.

Lastly, this work would like to serve as a stimulus to encourage further research in this interdisciplinary inquiry. Additional ways to efficiently deal with the challenges of machine learning should be contrasted, and special attention should be paid to the inefficiencies that externalities and monopolistic structures may generate.

## Data Availability

Not applicable.

## Appendix A

To incentivize the optimal level of interpretability and maximize aggregate social welfare, the following liability policy is proposed:

1. The default liability regime is fault-based, and the default burden of proof relies on the plaintiff.
2. The burden of proof may be alleviated if (1) the system is too complex to prove causation or fault by the plaintiff without incurring in unduly costs, (2) the machine learning model has been proven to cause damage in a similar situation, or (3) the probability that the model contributed to the harm is high [64].
3. Strict liability should be applied in the cases in which machine learning-powered solutions are operated in non-private environments and may typically cause significant harm [64].

This liability policy proposal, together with the power of demand-side market solutions, are expected to maximize aggregate social welfare without the need to explicitly limit any machine learning application of promulgate a right to explanation and to contest. Tort law and the pressure of consumers and voters are expected to sufficiently align the incentives of the organizations developing and operating machine learning models so that both present and future use cases generate more value than costs. Moreover, it is expected to foster innovation in the field of machine learning by balancing the right to compensation of consumers and the third parties affected with the need to prove causation or fault.

It is important to highlight that the objective should not be to minimize the risk of damages or to ensure that the totality of victims receive compensation, which entails an opportunity cost; instead, the objective should be to guarantee that the aggregate net effect is positive and the largest possible [30]. Nevertheless, there is still uncertainty regarding the extent to which this technology is going to produce significant damage and how this liability regime would cope with such cases in practice. Thus, any liability policy is going to require the monitoring of its economic effects and, potentially, modifications in the future.

## Appendix B

To ensure an optimal level of fairness in machine learning models while protecting minority groups in the relevant cases, two policies are proposed: (1) the publication of industry standards and good practices regarding algorithmic fairness and (2) a modification of anti-discrimination law procedures to accommodate the specificities of machine learning models.

It has to be pointed out that, even though it is likely that some of the deployed models will remain biased even with these measures in place, these cases will be only those without enough relevance to trigger legal claims. In other words, an optimal equilibrium of the social welfare–fairness trade-off is expected.

### Appendix B.1. Industry Standards and Good Practices

In order to provide legal certainty to organizations about the practices that are expected to be performed during the development and deployment of machine learning models to reduce the presence of biases, the publication of a globally harmonized set of industry standards and good practices is recommended.

Regarding their content, the following practices are proposed:

1. Form development and maintenance teams that are demographically diverse and interdisciplinary as is reasonably possible. This way, the likelihood of overlooking a source of bias is reduced thanks to the different sensibilities and expert knowledge in data science, the field of deployment, and ethics [79].
2. Track the lifecycle of the training dataset, including the methods used to collect the data, the way they were cleaned, and the individuals involved in the process, to minimize the introduction of any kind of adulteration [79].
3. Release of a trial model to study its behavior before full deployment in the wild. In other words, practice fairness forensics [79].
4. Reasonably use of state-of-the-art bias identification and removal techniques.
5. List and compare the relevant fairness metrics for the task at hand and the cost that they may represent in terms of predictive ability. Enumerate the reasons for choosing a specific metric if required.
6. Reasonably check the availability of alternative methods to perform the task at hand and compare their performance, cost, and fairness with the ones observed in the machine learning model being developed.
7. Periodically perform the activities outlined in this list and update the machine learning model if required. In other words, conducting continuous surveillance is a good practice.

These recommendations have a voluntary nature because machine learning has an almost endless amount of different use cases with their own specificities, which makes it impossible to determine suitable rules for each of them. Moreover, some use cases might not be relevant or profitable enough to require a strict adherence to them. This is key to avoid sub-optimally reducing the value generated by this technology by rendering some socially neutral use cases unprofitable.

Lastly, it is important to highlight that these industry standards and good practices should be periodically updated on the basis of the availability of new techniques ready for mass adoption produced by research in the field of data science.

### Appendix B.2. Modification of Anti-Discrimination Law Procedures

In order to palliate the hurdles that current anti-discrimination laws are expected to encounter when dealing with cases related to potential unfair machine learning models, the following modifications are proposed:

1. Create or hire groups of independent machine learning experts responsible for auditing the suspicious machine learning models and deliver a report to the jury. Auditors should (1) review the functioning of the machine learning model, (2) determine if the development of the model reasonably followed industry standards and good practices, and (3) recommend modifications, if applicable.
2. Train judges to facilitate their understanding and interpretation of the audit results to be able to produce sentences that optimize the social welfare–fairness trade-off.

Thus, the proposal is based on the professionalization of the judicial system so that it is able to properly examine this kind of cases.

Still, some clarifications are required:

- First, both judges and auditors must protect all the confidential information used to determine the sentence to avoid a depreciation of the trade secrets of the companies being audited. This is key to avoid harming their incentives to innovate and the security of the models (see Section 6).
- Second, auditors must be held accountable for the content of their reports, as usual. Any negligence in the review of the machine learning models should be penalized. This way, they have skin in the game and their incentives are aligned with those of the judicial system.
- Third, when determining the fairness of any machine learning model, juries should consider not only the compliance of the industry standards and good practices, as well as the observed fairness, but also the expected performance of the best available alternative, the associated short and long-term social welfare–fairness trade-offs, and the specificities of the context in which the model is being employed. This is of capital importance to decide which fairness metric should be optimized and whether the model is sufficiently fair to be used. Based on all these factors, the jury may deem the model as fair enough, ask for the remediation of some pitfalls, or decide to forbid its use until technology allows for solutions to its flaws, in addition to the corresponding fines, if applicable.
- Fourth, legal costs should be paid by the party that had their claims turned down, unless the jury considers that the case was sufficiently doubtful and critical to raise a legal claim and the plaintiff is in a situation of vulnerability. This is a key feature to optimize the utilization of this mechanism, which makes use of the limited public resources.

In sum, the proposed policies aim at providing legal certainty and aligning the incentives of all the parties towards the optimization of the social welfare–fairness trade-off. First, organizations have additional incentives to follow the industry standards and good practices and consider algorithmic fairness when developing and deploying sensitive machine learning models. This happens because they anticipate that they could be penalized. Second, citizens have the ability to pursue legal claims when they identify reasonable doubts of fairness in relevant machine learning applications. Third, auditors are incentivized to provide an objective review of the models and keep trade secrets. Lastly, judges are able to properly evaluate these cases to optimize the social welfare–fairness trade-off.
